# Genetic inbreeding depression load for conformation defects and dressage traits in the Pura Raza Española horse

**DOI:** 10.5713/ab.25.0362

**Published:** 2025-10-22

**Authors:** María Ripollés-Lobo, Davinia Isabel Perdomo-González, Pedro Azor, Mercedes Valera

**Affiliations:** 1 Departamento Agronomía, Escuela Técnica Superior de Ingeniería Agronómica, Universidad de Sevilla, Sevilla, Spain; 2 Departamento Producción Animal, Facultad de Veterinaria, Universidad Complutense de Madrid, Madrid, Spain; 3 Real Asociación Nacional de Criadores de Caballos de Pura Raza Española, Sevilla, Spain

**Keywords:** Equine, Genetic Evaluation, Heritability, Inbreeding Depression, Partial Inbreeding

## Abstract

**Objective:**

Investigate the genetic *inbreeding depression load* (IDL) on two dressage traits and two conformational defects in horses.

**Methods:**

The dataset included performance records for *Walk* and *Points per Reprise* (PPR) (N:43,838) and conformation data for *Closed* and *Convergent hocks* (N:57,949). Pedigree information spanned over 400,000 individuals. *Inbreeding coefficients* (F, F_6_, F_k_) and partial inbreeding coefficient (F_ij_) were computed to quantify the IDL using Bayesian approach. Linear and ordinal logistic regressions were used to assess the relationship between inbreeding and phenotypic values. Additionally, Pearson correlations were computed to explore IDL relationships across traits.

**Results:**

Significant inbreeding depression was detected for *Walk* and PPR across all inbreeding coefficients, with stronger effects for recent inbreeding (F_6_). *Closed hock* showed significant positive associations with inbreeding, while *Convergent hock* displayed mixed responses. Heritability estimates were low for gait traits (0.02) and moderate for defects (0.15–0.22). Only a small percentage of animals exhibited favorable IDL values for a 10% inbreeding: 2.94% (*Walk*), 0.77% (PPR), 1.30% (*Closed hock*), and 0.69% (*Convergent hock*). While heritability reflects the variation actually observed within a population, IDL ratios indicate the possible variation that exists, regardless of whether it is currently expressed or not. Pearson correlations revealed moderate positive IDL associations between *Walk* and PPR (0.45), and lower correlations between gait and defect traits.

**Conclusion:**

Results highlight the importance of modeling individual-specific IDL in Pura Raza Española genetic evaluations. IDL explained a substantial proportion of phenotypic variance and showed trait-specific patterns. Incorporating IDL into selection strategies enables more informed breeding decisions, allowing the retention of valuable genetic lines while minimizing deleterious inbreeding effects, thereby supporting more sustainable and resilient genetic improvement.

## INTRODUCTION

Inbreeding depression is a well-known biological phenomenon resulting from matings between genetically related individuals. The increase in homozygosity also increases the expression of deleterious recessive alleles while heterozygosity advantages are reduced [1]. In domestic animals, inbreeding depression has been shown to negatively affect fitness-related traits, including fertility, survival, body development, and functional performance [2,3]. These effects are of particular concern in closed populations subject to intensive selection pressure.

The Pura Raza Española (PRE) horse is a closed population, managed under a closed Studbook since 1912 [4]. Modern PRE horses are selected for both dressage aptitude and morphological traits aligned with breed standards, which has contributed to a strong fixation of type.

Several studies have reported evidence of inbreeding depression in morphological traits across horse breeds, including PRE [5,6]. In the PRE breed, the potential impact of inbreeding on dressage traits is still underexplored, but recent studies have highlighted the usefulness of modelling *inbreeding depression load* (IDL) to evaluate susceptibility in both morphological and functional traits [4,6]. Traditionally, Wright’s inbreeding coefficient (F) has been the standard for estimating individual inbreeding levels. However, it fails to account for the origin of inbreeding or the specific contribution of different ancestors. To overcome this limitation, García-Cortés et al [7] proposed the use of partial inbreeding coefficients (F_ij_) by decomposing F into its ancestral sources. Later, the use of F_ij_ allowed the estimation of the individual IDL, successfully applied in PRE to reproductive and morphological traits, offering more informative tools to detect animals with high genetic value but reduced susceptibility to inbreeding depression [4,6,8].

Furthermore, complementary pedigree-based metrics such as recent inbreeding (F_6_–over six generations) and ancestral inbreeding (F_k_–Kalinowski inbreeding coefficient) provide additional information on the timing and persistence of inbreeding effects [9–11]. These indicators are particularly important in structured or subdivided populations like the PRE, where historical bottlenecks, line breeding, and founder imbalance have shaped the genetic architecture [12]. Evaluating the genetic IDL for both dressage traits and conformational defects in PRE horses using F_ij_ may provide useful insights for genetic evaluation and sustainable breeding strategies.

Therefore, the objectives were: (1) to determine the presence and magnitude of inbreeding depression in these traits using different inbreeding estimators; (2) to estimate genetic parameters for selected dressage traits and conformational defects in PRE horses; and (3) to estimate the individual IDL to support the selection of robust and genetically valuable individuals.

## MATERIALS AND METHODS

Due to their relevance in the functional and morphological evaluation of PRE horses, the variables studied were:

- Dressage variables:
  • *Walk*: One of the horse’s three natural gaits. In the classical dressage, it is evaluated based on its regularity, rhythm, and impulsion. The score assigned to this gait serves as a functional indicator in aptitude tests and competitions [13].
  • *Points per Reprise* (PPR): A composite measure reflecting the horse’s overall performance in dressage competitions, integrating scores obtained across various gaits and exercises.
- Hock conformation defects (Table 1):
  • *Closed hock*: Conformational defect observed in the lateral view, where the leg forms an excessively closed angle with the cannon bone [14].
  • *Convergent hock*: Conformational defect observed in the rear view, where the hocks deviate inward, drawing closer to each other and straying from the aplomb line [14].

The dressage dataset comprised a total of 43,838 performance records from 4,546 PRE horses (4,221 males and 325 females), collected between 2004–2023 during dressage competitions. In these events, the scoring system utilized the average of three judges’ evaluations for the *Walk* movement, which was assessed on a continuous scale from 1 (poor quality) to 9 (excellent quality). Additionally, the PPR was recorded as a composite score summarizing the overall performance of the horse-rider combination throughout the entire dressage test. For this purpose, judges individually scored each movement of the test on a scale from 0 (not executed) to 10 (excellent execution). These scores were then weighted according to the relevance of each movement, and their weighted average was calculated. This final average was subsequently multiplied by 10 to obtain the final score, expressed on a continuous scale ranging from 10 (poor quality) to 100 (excellent quality). Although the score of each judge individually was not available, but rather the average score of the 3 judges, notably a key strength of the dataset lies in the interconnection of competitions through shared judges, as many of them officiated at multiple events. This overlap created a robust network of evaluations, enabling the modelling of judge effects across competitions and improving the consistency and reliability of the evaluations.

The defects traits dataset included conformation data from 57,949 horses (19,448 males and 38,501 females). The evaluations were performed by a group of 12 specially trained veterinarians who routinely conduct the basic aptitude tests for this breed. The phenotypic assessment of conformational defects was carried out while the horse stood on a hard, level surface, in a natural stance. Horses were positioned with their forelegs and hindlegs parallel and as perpendicular as possible, with hooves properly aligned. No sedatives were administered during the evaluation process. Hock conformational defects were assessed using a linear scoring scale from 1 to 3. Class 1 corresponds to the absence of the defect; class 2 corresponds to the slight presence of the defect and class 3 is the most pronounced degree of defect.

To ensure the consistency of the analysis, only horses with phenotype and both parents registered were included. Pedigree information was sourced from the Royal Association National of Purebred Spanish Horse Breeders (ANCCE) studbook. Based on the selected individuals, a pedigree comprising 398,866 horses (194,911 males and 203,955 females) was constructed with all the known generations.

*Wright’s coefficient of inbreeding* (F), also referred to as classical inbreeding, was calculated as the probability that an individual carries two *alleles identical by descent* (IBD) at a randomly selected locus. F and *coefficient of inbreeding up to the sixth generation* (F_6_) were estimated following the methodology of Meuwissen and Luo [9], using the Endog v4.8 software [15]. The *ancestral inbreeding coefficient proposed* by Kalinowski (F_k_), defined as the probability that an allele is currently autozygous and has been IBD in at least one previous generation [10], was computed using GRain 2.2 [11], a coefficient recently linked to inbreeding depression in this breed [4].

Moreover, F_ij_, representing the joint probability that an individual *i* is autozygous for an allele inherited from a specific ancestor *j* [16,17], were determined for all individuals in the population, based on the methodology described by Casellas [18]. This strategy enabled the partitioning of total inbreeding into contributions derived from the coancestry between each individual’s parents, taking into account both the original founders and the Mendelian sampling variance among non-founders [7,19]. As a result, 193,214 F_ij_ coefficients exceeding 0.00001 were identified, corresponding to 3,994 common ancestors (including both founders and non-founders from paternal and maternal lines). Each of these ancestors contributed F_ij_ values to between 14 and 102 descendants. To assess the effect of inbreeding depression, simple linear regression coefficients were estimated between the inbreeding coefficients (F, F_6_, and F_k_) and the phenotypic values of the analysed traits. Two regression models were employed using R. The first, based on the *lm()* function, fits continuous response variables using ordinary least squares under the assumption of normally distributed errors (used for *Walk* and PPR). The second, utilizing the *polr ()* function from the MASS package, is tailored for ordered categorical response variables by estimating the probability of the response falling at or below a given category in accordance with the proportional odds assumption (applied to *Closed* and *Convergent hocks*). The t-values resulting from these regression models serve as indicators of the statistical significance of each predictor, with higher absolute t-values denoting a stronger contribution of the corresponding variable to the model. All statistical analyses were conducted using the R software [20].

For the genetic parameters estimation, each trait (*y*) was analysed using a Bayesian linear model initially proposed by Casellas [18] and later reformulated by Varona et al [8]. This model incorporates both the standard breeding value and the IDL attributable to the individuals’ ancestors as random genetic effects, capturing the phenotypic variation while accounting for the additive nature of the individual IDL (i). The final model applied to dressage traits was expressed as:


(1) y=fc+Xb+Wh+Rr+Zu+Ki+e

The model used for defect traits followed a reduced form, represented as:


(2) y=fc+Xb+Zu+Ki+e

These models incorporate a vector representing the total inbreeding of the evaluated individual (f), a covariate associated with the overall inbreeding depression (c), systematic effects (b), infinitesimal additive genetic contributions (u), individual IDL effects (i), and residual terms (e). In addition, gait models included the permanent environment effect (h) and the rider random effect (r). The prior distributions assumed for the additive genetic effects, the IDL effects, and the residuals were as follows:


(3) $$\left(\begin{array}{c} u\\ i \end{array}\right)˜\text{N}\left(\begin{array}{c} 0\\ 0 \end{array},G⊗A\right),\text{h}˜\text{N}\left(0,\sigma_{\text{h}}^{2}\right),\text{r}˜\text{N}\left(0,\sigma_{\text{r}}^{2}\right),\;\text{and e}˜\text{N}\left(0,\text{I}\sigma_{\text{e}}^{2}\right),$$

where $\text{G}=\left(\begin{array}{cc} \sigma_{u}^{2} & \sigma_{ui}\\ \sigma_{ui} & \sigma_{i}^{2} \end{array}\right)$;

A is the numerator of relationship matrix; $\sigma_{u}^{2},\sigma_{i}^{2}$, σ_h_^2^, σ_r_^2^ and σ_e_^2^ are the associated variance components for additive, IDL, permanent and rider effects, and σ*_ui_* is the covariance between the additive genetic and the IDL effects, respectively. R, W, X, and Z are incidence matrices of rider and permanent effects, systematic effects and additive genetic contributions, respectively, and K = T(I−P). T is a lower triangular matrix in which each non-zero element is a F_ij_ which links the phenotype of an inbred individual to each ancestor causing inbreeding. For computational reasons, each F_ij_ was multiplied by 10 to obtain the IDLvariance for a F_ij_ of 10%. P is a projection matrix with a 0 diagonal and 0.5 in the elements that link an individual to its sire and dam, and I is the identity matrix.

Specifically, for dressage traits, the systematic effects vector (b) included the fixed effects of sex (2 levels), stud size (3 levels: small, fewer than 3 foals born per year; medium, between 3 and 9 foals per year; and large, more than 9 foals per year), location-date of competition (932 levels), and competition level (11 levels). Rider (1,395 levels) and permanent environmental effects (3,638 levels) were included as random effects, and evaluation age was incorporated as a covariate. For conformational defect traits, the model was simplified and only included sex and stud size as fixed effects, with evaluation age also included as a covariate. The qualifying effect was not included since it was not significant (data not shown), as had already happened with this type of variables.

The *Markov chain Monte Carlo* (MCMC) model was implemented using custom software developed in FORTRAN90 [8]. Convergence was assessed by visual inspection of trace and running mean diagnostic plots; no trend was observed after burn-in. A single chain consisting of 1,000,000 samples, following a burn-in period of 20,000, was used for each trait. This analysis further enabled the calculation of the IDL ratios, which can be interpreted as the proportion of the variation in each phenotypic unit attributable to the variation in inbreeding depression effects. This proportion is estimated in a theoretical population where each individual possesses a partial inbreeding coefficient derived from a single, specific ancestor [6,8]. Inference was based on posterior distributions and HPD95 credible intervals; therefore, classical frequentist residual diagnostics (e.g., QQ-plots, Shapiro–Wilk) are not required within this Bayesian framework. Finally, to assess the relationships among IDL among traits, Pearson correlation coefficients were calculated using R software [20] and the *cor()* function with the Pearson method.

To evaluate the potential impact of incorporating IDL into selection decisions, a series of selection index framework were implemented following Hazel and Lush [21], adapted for estimated breeding values (EBV) as described by Gutiérrez et al [22]. Three index scenarios were simulated: using the EBV selection criteria as the selection objective (index 1), using the IDL as selection criteria and the EBV as the selection objective (index 2), and combining the EBV and the IDL as selection and criteria objectives (index 3). For each index, expected genetic response (EGR) were computed, assuming equal additive genetic variance (unit scale), selection intensity of 1, and the same heritability-based accuracy across individuals [23]. Different economic weight vector (p′) were tested for index 3.

## RESULTS

The descriptive statistics for traits analysed in the PRE population can be seen in Table 2. For the continuous gait traits, *Walk* has a mean of 6.67 with a very low standard deviation (0.005) and a coefficient of variation (CV) of 0.07%, indicating low dispersion relative to its mean. In contrast, PPR exhibits a mean of 65.84 with a standard deviation of 5.22 and a CV of 7.91%, showing a slightly lower relative variability compared to *Walk*. The range for *Walk* spans from a minimum of 1 to a maximum of 9, and for PPR from 11.85 to 98.10. Regarding the defect traits, which are assessed on an ordinal scale where lower scores indicate better conformation, both *Closed* and *Convergent hocks* are confined to a range of 1 to 3. For *Closed hock*, the mean is 1.32 with an SD of 0.60. The distribution is skewed toward the absence of the defect, with 74.76% of animals scored as 1 and 25.24% showing the presence of the defect (class 2+3). In contrast, *Convergent hock* displays a higher mean score of 1.93 with a standard deviation of 0.68. Its distribution is less favourable, with only 26.91% of animals scored as 1 and 73.10% showing the presence of the defect (class 2+3).

The regression coefficients (b) for dressage and defect traits with the different inbreeding coefficients in the PRE horse are displayed in Table 3. For the gait trait *Walk*, negative regression coefficients were observed across all inbreeding measures. Specifically, the coefficients for F, F_6_, and F_k_ were −0.68, −0.74, and −0.75, respectively. These coefficients were highly significant (all p<0.001). Similarly, the PPR trait exhibited significant negative associations with inbreeding, with coefficients of −3.5 for F, −4.08 for F_6_, and −3.10 for F_k_. For the defect trait *Closed*, the ordinal logistic regression revealed positive coefficients for all inbreeding measures, evidencing inbreeding depression: 1.60 for F, 1.95 for F_6_, and 2.18 for F_k_. These values were statistically significant. *Convergent hock* trait presented a more variable pattern. The regression coefficient for F was not statistically significant, whereas F_6_ yielded a positive coefficient of 0.43 and F_k_ produced a significant negative coefficient of −0.67.

Table 4 shows heritability estimates, IDL ratios (d^2^) and the variances attributed to the direct additive genetic effect, IDL (corresponding to an inbreeding value of 10%), the random effects (for dressage traits only) and the residual effect of the analysed traits. Analogously to heritability, d^2^ were calculated as the relative magnitude of the IDL variances. The heritability estimates were 0.02 both for *Walk* and PPR, while higher values were obtained for *Closed hock* (0.15) and *Convergent hock* (0.22). d^2^ calculated for an inbreeding value of 10%, were higher than heritability values for all traits (0.81, 0.89 and 0.21 for *Walk*, PPR and *Closed hock*) except for *Convergent hock* (0.18). The IDL variances were higher than the direct additive genetic variances as a direct consequence of the arbitrary scaling of F_ij_ by 10. Finally, as expected, repeatability (r) slightly exceeded heritability (h^2^) for the dressage traits with repeated records (*Walk* r: 0.05 vs h^2^: 0.02; PPR r: 0.04 vs h^2^ = 0.02; Table 4).

**Figure 1:**
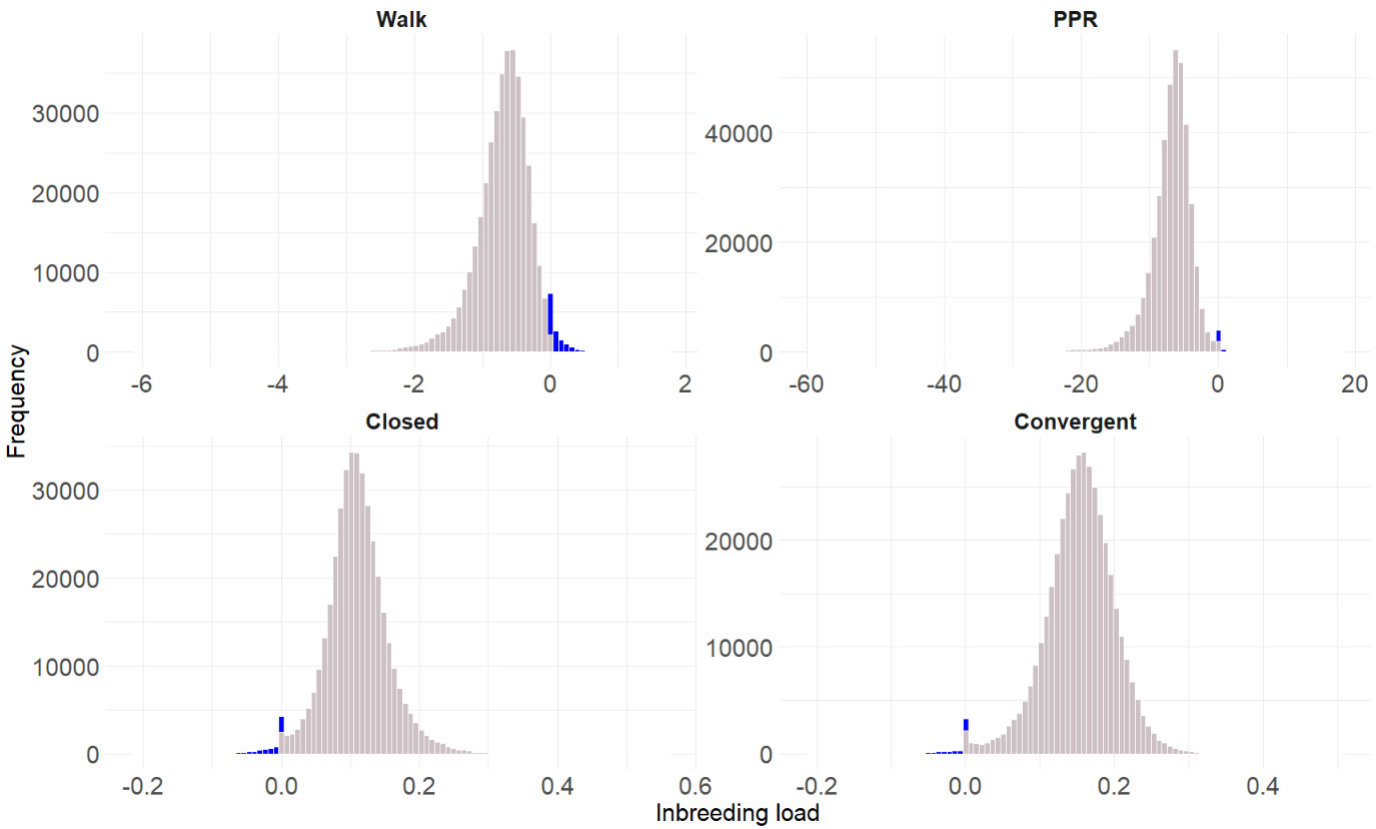
Inbreeding depression load values frequency for all the analyzed traits in the Pura Raza Española horse population evaluated. In blue, inbreeding depression load values under or upper, depending on the trait, the regression slope between inbreeding depression load values with the inbreeding (for a 10% of inbreeding). PPR: total points per reprise.

Figure 1 displays histograms of the estimated IDL for each of the four traits within the evaluated PRE horse population. Across all traits, the distributions are largely unimodal and centered around zero, indicating that most individuals exhibit moderate IL. However, a subset of horses displays more extreme positive or negative values. The blue bars highlight IDL values surpassing (or falling below) the predicted regression slope threshold for an assumed 10% inbreeding level. Notably, these regions suggest that only a small fraction of the population experiences substantially higher or lower IDL which implies that the use of these animals as common ancestors of the future animals will contribute to an improvement of those traits even when they transmit a high inbreeding level. Specifically, the predicted regression slope threshold between IDL and the F for an assumed 10% inbreeding level were −0.01 (*Walk*, *Closed hock* and *Convergent hock*) and −0.02 (PPR), implying that the proportion of animals that can be used as a common ancestor with positive results are low, 2.94% (*Walk*), 0.77% (PPR), 1.30 (*Closed hock*) and 0.69% (*Convergent hock*).

Table 5 shows the Pearson correlation coefficients between IDL values and the number (and percentage) of individuals exhibiting simultaneous improvement in IDL values for each pairwise combination of traits. The correlation coefficients indicate a moderate positive relationship between *Walk* and PPR (0.45). The correlations between *Walk* and *Convergent hock* (0.07), and between *Walk* and *Closed hock* (0.07), as well as those between PPR and the defect traits, *Closed hock* (−0.02) and *Convergent hock* (0.06), indicate only a weak relationship.

Regarding the number and percentage of animals showing coincident favourable IDL across traits, the largest overlap was again between *Walk* and PPR, with 2030 animals (51%). In contrast, only 16% to 32% of the population showed overlapping improvement between one gait trait and one defect trait, and the lowest overlap was found between PPR and *Convergent hock* (16%).

**Figure 2:**
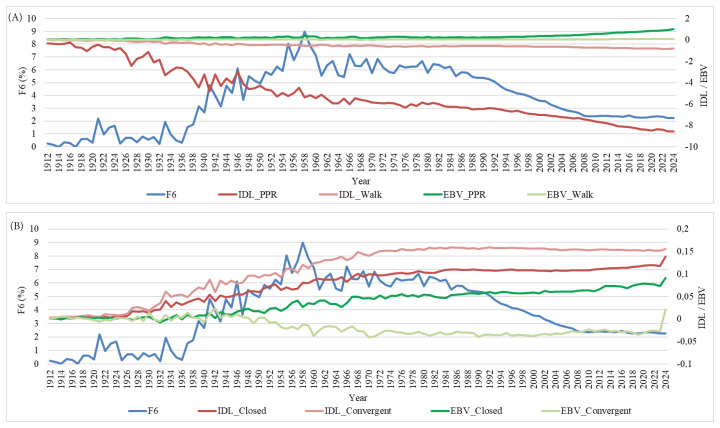
Temporal trends of recent inbreeding, breeding values, and IDL for functional variables (A) and conformational variables (B) in the Pura Raza Española horse. Favourable direction: IDL higher for dressage, lower for defects; EBV higher for dressage, lower for defects. F_6_, recent inbreeding; IDL, inbreeding depression load; EBV, estimated breeding values.

Beyond these cross-trait overlaps, Figure 2 summarizes the temporal trajectories of F_6_, EBV, and individual IDL by cohort. We focused on F_6_ as the time-varying metric because it captures recent inbreeding and showed the strongest and most consistent associations with the phenotypes in our regressions (Table 3). F_6_ increased from the early decades of the twentieth century, peaked around 1958, and then declined while remaining above early-cohort levels. Over the same period, mean EBV for dressage traits remained broadly stable, with slight improvements in recent decades, whereas IDL for PPR decreased (became more negative) and IDL for *Walk* remained essentially stable. For the hock defects, IDL increased over time for *Convergent hock*, while EBV for *Closed hock* stabilised with slight recent improvements and EBV for *Convergent hock* showed an overall decline.

The expected EBV responses for *Walk* and *Closed hock*, together with the relative change versus Index 1 (baseline = 100%), are shown in Table 6. Index 1 (EBV-only) delivered the largest response for both traits (0.14 for *Walk*; 0.39 for *Closed hock*). Index 2 (IDL-only) produced a marked reduction in EBV gain, especially for *Walk* (−589.46%). Hybrid indices that combine EBV and IDL (Index 3 variants) yielded progressively lower EBV responses as the IDL weight increased.

## DISCUSSION

Descriptive results (Table 2) show a distribution typical of intensively selected populations, such as the PRE breed. The average *Walk* score (6.67) is like that of other dressage breeds, but its low variability (0.07%) suggests possible genetic fixation due to selection. This might also reflect a tendency among judges to favour intermediate scores, limiting the use of extreme values and contributing to score homogenization. In contrast, Warmblood horses show similar mean *Walk* scores but with variation coefficients above 15%, indicating more genetic variability [24,25]. The PPR had a mean of 65.84 and a moderate SD (5.22). Sánchez-Guerrero et al [26] reported a nearly identical average (65.33) and a CV of 6.94% in PRE horses, supporting trait consistency. While phenotypic variability in PPR appears stable across PRE studies, comparisons with other breeds are scarce. However, Solé et al [27] revealed that Warmblood horses, particularly those competing at the international level, tend to outperform both Lusitano and PRE horses in dressage disciplines.

For conformational defects, most animals were unaffected by for *Closed hock* (74.76%), but only 26.91% were unaffected for *Convergent hock*. These results match those by Ripollés-Lobo et al [14], who found 77.91% and 25.88%, respectively. This confirms the high frequency and functional relevance of *Convergent hock* defects in PREs. Compared to other breeds, the PRE shows intermediate prevalence. In Menorca Purebreds, Ripollés-Lobo et al [28] found fewer healthy animals for *Closed hock* (37.47%) and more for *Convergent hock* (61.17%).

Inbreeding depression has been studied in different horse breeds [5,29–31]. The magnitude of inbreeding depression is influenced not only by the overall inbreeding level but also by the timing and origin of inbreeding, which can be better captured through refined coefficients such as F_6_ and Fk, rather than classical *Wright inbreeding coefficient*.

Comparatively, the magnitude of IDL and the negative regressions with recent inbreeding observed here align with inbreeding-depression evidence on performance in other sport breeds (notably Thoroughbreds) and with meta-analytic results across livestock [32,33]. However, the particularly strong response in PPR in PRE is consistent with a closed studbook and the historical concentration of elite sires [4]. In Lusitano horses, heritabilities for dressage and gait traits are typically higher [34], suggesting a lower expressed recessive load for these traits. Within PRE, morphological traits and fertility show detectable inbreeding effects but different sensitivities and responses to selection [6,31].

For the functional traits (*Walk* and PPR) all three inbreeding coefficients (F, F_6_, F_k_) showed consistent negative associations, suggesting that higher levels of inbreeding are linked to poorer performance in dressage-related abilities (Table 3). This agrees with prior studies on gait and locomotion traits across breeds like Andalusian horses [5]; Lusitan horses [35]; Thoroughbred horses [29,32]. The most pronounced effects occurred with F_6_, supporting the idea that recent inbreeding increases the likelihood of phenotypic expression of deleterious recessives, as these have not yet been purged from the genome. This interpretation aligns with findings in Thoroughbred horses by Hill et al [32], who identified a significant association between a specific haplotype on chromosome ECA14 (THR14) and reduced probability of racing. In comparison, the weaker or even inverse regressions observed with F_k_, in PPR and *Convergent hock*, suggest that ancestral inbreeding may be associated with partial purging of harmful alleles, especially in lines subjected to consistent selection pressure. This is supported by findings in other horse breeds as Lusitano horses [34], where ancestral inbreeding effects were less pronounced and often curvilinear.

Todd et al [29] reinforced this interpretation using the ancestral history coefficient (AHC) and F_k_. While global inbreeding reduced performance, ancestral inbreeding improved it, and founder-specific effects highlighted the influence of inbreeding origin. Casellas [18], proposed a similar framework, noting that inbreeding depression depends on which genomic regions and ancestors are involved, not just on overall F.

Morphological defects examined, exhibited more heterogeneous responses. *Closed hock* showed a consistent positive regression with all inbreeding coefficients, suggesting the persistence of a stable additive load that has not been effectively purged over time. These results agree with Ripollés-Lobo et al [28] in Menorca Purebred. On the other hand, *Convergent hock* presented a distinct and biologically meaningful pattern, while the regression with F_6_ was positive, indicating ongoing inbreeding depression, the association with F_k_ was negative, supporting a purging effect in animals with a history of ancestral inbreeding. This duality reinforces the idea that visible, easily penalized traits may be subjected to more effective purging dynamics, as suggested by Casellas [18]. This pattern has also been confirmed in the PRE population by Ripollés-Lobo et al [14], who found that animals with F>12.5% were significantly more likely to express limb defects, particularly *Convergent hock*, which also had a high prevalence (74.1%), yet many severely affected animals may be excluded from registration, masking the full impact of inbreeding.

The idea that not all conformation traits are equally sensitive to inbreeding is further supported by Vostrý [36] who analysed 22 linear conformation traits in Czech cold-blooded horses and found variable but detectable inbreeding effects, even at low mean F. Traits related to movement and overall body size were more affected by inbreeding than those associated with specific anatomical regions, such as localized limb structures. Moreover, they demonstrated that including inbreeding in the model altered heritability estimates and breeding value reliability, underscoring the relevance of modelling inbreeding explicitly in genetic evaluations.

The large-scale meta-analysis by Doekes et al [33] across seven livestock species showed that inbreeding depression is pervasive across all trait types, not just those related to fitness. Their study emphasized that refined metrics such as F_k_ or runs of homozygosity (ROH) improve the estimation of inbreeding effects and that even moderate inbreeding levels can significantly impact trait means and variances, especially in closed populations.

Heritability estimates for *Walk* and PPR were low (Table 4), contrasting with higher values reported by Sánchez-Guerrero et al [26] in the same breed (0.21 for *Walk*, 0.30 for PPR). These differences could be due to variations in data structure, model specification, or trait definition. In other breeds, *Walk* heritability ranges from 0.08 to 0.38, and PPR from 0.18 to 0.32 (Dutch Warmblood [37]; Hungarian sport horse [38]; Finnhorse and Standardbred foal [39]; Lusitano horse [35]). Heritability of hock defects varies by breed and method used. In PRE horses, Ripollés-Lobo et al [14] reported moderate to high values (*Closed hock*: 0.26; *Convergent hock:* 0.42) (Table 4), while in Menorca horses (0.12 and 0.24, respectively) [28]. The very low heritability estimates for *Walk* and PPR (0.02), together with the high d^2^ ratios (0.81–0.89), are primarily a consequence of the modelling strategy adopted here. Unlike earlier PRE analyses, our Bayesian framework explicitly partitions the genetic variance into a direct additive component and an individual-specific IDL; accordingly, variance that in additive-only models would inflate heritability is reallocated to the IDL term, yielding lower apparent heritability values. This conceptual partitioning, rather than an abrupt biological change, likely explains most of the discrepancy with previous PRE reports [26,40]. Additional contributors may include differences in data structure and model specification (wider time span and stronger control of rider and permanent environmental effects), as well as judge-based subjectivity and environmental heterogeneity related to training level, all of which can further reduce the detectable additive genetic signal relative to studies in PRE or Warmblood populations [25,34,37].

The results highlight the relevance of IDL in the genetic architecture of performance and defect traits in PRE horses (Table 4). In *Walk*, PPR, and *Closed hock*; IDL variance exceeded additive genetic variance, with only *Convergent hock* showing higher additive values. This pattern aligns with findings in cattle [8] and equines [4,6], supporting that the transmitted recessive genetic load can explain a larger share of the phenotypic variance than additive effects alone, especially when partial inbreeding (F_ij_) is considered.

The d^2^, representing the proportion of phenotypic variance due to inbreeding load (assuming 10% F_ij_) was particularly high for dressage traits: 0.81 for *Walk* and 0.89 for PPR, far exceeding their heritabilities (both 0.02). This indicates that most genetic variation in these traits originates from inbreeding effects rather than additive contributions. These high ratios are not only biologically meaningful but also potentially actionable, as they provide insight into how specific ancestral lineages might affect phenotypes through inbreeding. These figures reflect the underlying genetic load that possibly will exist in the population due to founder effects and pedigree bottlenecks, despite moderate additive control. Such results echo Casellas [18] who highlighted the importance of individual-specific IDL in identifying deleterious recessive variance often missed by the additive models.

Figure 1 illustrates the complexity of inbreeding depression through individual-level IDL distributions for each trait. All traits show roughly unimodal, symmetrical distributions cantered around zero, as also reported by Casellas [18], Poyato-Bonilla et al [6] and Perdomo-González et al [4]. This suggests most animals will have an intermediate IDL, although a subset displays extreme values, either positive or negative. Individuals with IDL values lower than the regression between IDL and the inbreeding are likely to transmit to their inbred offspring a potential overcome the negative effects of inbreeding on defects traits. Conversely, individuals with IDL values higher than the regression between IDL and the inbreeding may transmit to their inbred offspring a potential to offset the negative effects of inbreeding on dressage-related traits. This observation is crucial because it challenges the classical assumption that inbreeding effects are uniformly detrimental and instead supports the concept of individual heterogeneity in IDL, as discussed by Varona et al [8] and Perdomo-González et al [4]. Moreover, the Figure 1 highlights thresholds of IDL corresponding to an F value of 10%, providing a benchmark to identify tolerant or even favourable transmitters of IDL. For instance, only 0.77% of individuals had an IDL above −0.02 for PPR, suggesting that an individual with a F_ij_ of 10% or more, derived from one of those specific common ancestors, tends to perform better in dressage than its contemporaries. This implies that only a small elite group of animals can improve PPR performance in inbred offspring, despite the high susceptibility of this trait to inbreeding depression. Only 0.69%–2.94% of animals exceed the favourable IDL threshold under an assumed 10% F_ij_, indicating that most lines still transmit a significant recessive load. This scarcity of resilient lineages (e.g., those predicted to perform well despite a 10% of inbreeding) is consistent with expectations from closed populations that have experienced historical bottlenecks and long-term overuse of a limited number of elite sires. Practically, this low resilience carries economic consequences, including reduced sporting performance and international competitiveness, and underscores the need to complement EBV based selection with IDL to preserve valuable lines while limiting the recessive load.

From a breeding perspective, these results provide a valuable tool: instead of discarding animals based only on inbreeding coefficients, breeders can select individuals with favourable IDL values (even at moderate inbreeding) preserving valuable lines while reducing harmful recessive effects [4,18].

Together, the results from Table 4 and Figure 1 provided strong evidence to include individual-specific IDL in PRE genetic evaluations. That IDL variance often exceeds additive variance, and that d^2^ surpasses heritability in key traits (PPR and *Walk*), clearly indicated the concealed but substantial influence of recessive load. The distribution and thresholds of IDL further offer breeders a practical way to identify inbred individuals that avoid expected phenotypic decline and may even enhance performance or reduce defects. This suggest that managing inbreeding based solely on pedigree coefficients (F, F_k_) may be insufficient. Selection based on IDL allows for more refined decisions, preserving valuable lines while limiting deleterious alleles, a scientifically grounded improvement to current PRE breeding strategies, particularly in traits with low heritability but high susceptibility to inbreeding depression.

Cross-trait correlations among IDL values were generally low to moderate and mostly positive (Table 5), consistent with some shared recessive burden across traits [33]. However, because the favourable IDL direction differs by trait (higher for dressage, lower for defects), a positive correlation does not necessarily imply a favourable correlated response. In practice, the proportion of animals showing simultaneous favourable IDL in both traits was limited, ranging from 16% to 32% across dressage–defect pairs and as low as 16% for PPR with *Convergent hock* (Table 5). These results support using IDL alongside EBV when ranking and mating, rather than relying on EBV alone.

As shown in Figure 2, despite the mid-century peak and later decline in F6, the recessive burden has not abated. In dressage, IDL for PPR became more negative, which is unfavourable because higher IDL denotes greater resilience to inbreeding, while IDL for *Walk* was essentially stable and EBV remained broadly stable with slight recent gains. For hock defects, IDL increased for *Convergent*, which is unfavourable since lower (more negative) IDL is desirable for defects, whereas EBV for *Convergent hock* declined, which is favourable, and EBV for *Closed hock* was largely stable with slight recent improvements; IDL for *Closed hock* showed no marked trend.

Two representative traits were selected based on the sign of their correlation between EBV and IDL for the selection index simulations: one with a positive correlation (e.g., *Closed hock*) and one with a negative correlation (e.g., *Walk*) (Table 6). For *Closed hock*, which shows a positive correlation between EBV and IDL, none of the designed indices appear to produce any improvement and index 1 will be recommended. In contrast, although a superficially similar pattern is observed for *Walk*, the negative correlation between EBV and IDL in this case results in a different outcome. The index 3 assigning 90% economic weight to EBV and 10% to IDL may be beneficial in the long term for *Walk*. Although it results in slightly lower genetic progress, it leads to an increase in IDL over time, which could be advantageous in inbred populations such as the PRE, by lowering the expected risk of inbreeding depression.

These patterns indicate trait-specific selection responses. Strong penalties and culling reduce the expression of *Convergent hock* (downward EBV), but the hidden recessive load persists (rising IDL), consistent with incomplete purging and with the negative F_k_ regression for *Convergent hock*. Conversely, the diffuse recessive burden in the polygenic and subjectively scored PPR proved difficult to remove. Practically, EBV only selection is insufficient; programmes should incorporate IDL aware decisions, for example EBV+IDL indices and IDL informed mate allocation, while diversifying lines and limiting the overuse of elite sires to curb the accumulation of recent inbreeding.

## CONCLUSION

The findings of this study underscore the value of incorporating Individual IDL into the genetic evaluations of PRE horses. By going beyond traditional additive genetic values, IDL captures the specific impact of deleterious recessive alleles transmitted through shared ancestry, offering a more comprehensive explanation of phenotypic variability in both functional traits (*Walk* and PPR) and morphological defects (*Closed* and *Convergent hocks*). In many cases, the genetic load associated with inbreeding accounts for a larger portion of this variability than additive effects alone, which is particularly relevant in inbred or semi-closed populations such as the PRE. The identification of individuals with favourable IDL values opens the door to more refined selection strategies that maintain genetic diversity while enhancing performance and reducing defects.

## Figures and Tables

**Table 1:** Graphic description of the hock conformation defects analysed in the Pura Raza Española horse population

Hock defect	Class		Hock defect	Class	
	
	1 (correct)	3 (evident)		1 (correct)	3 (evident)
Closed	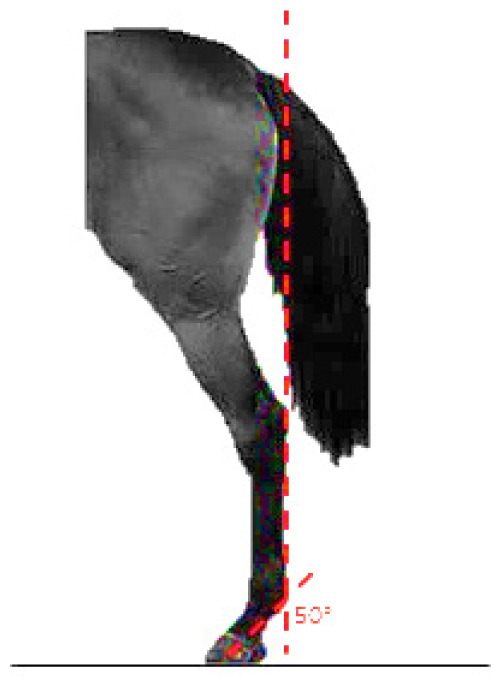	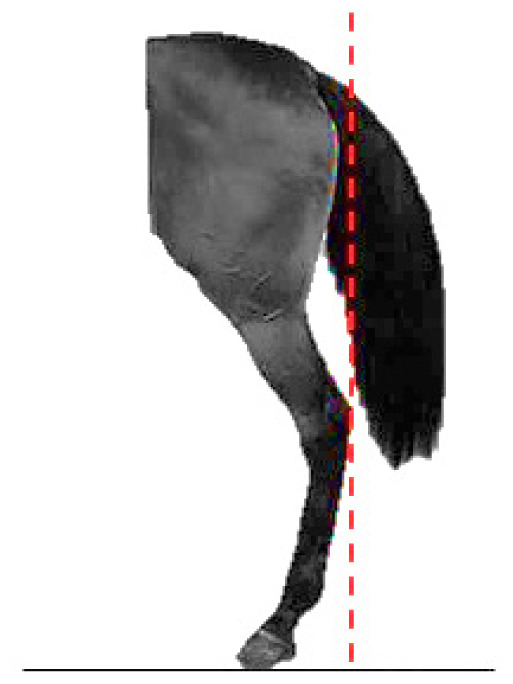	Convergent	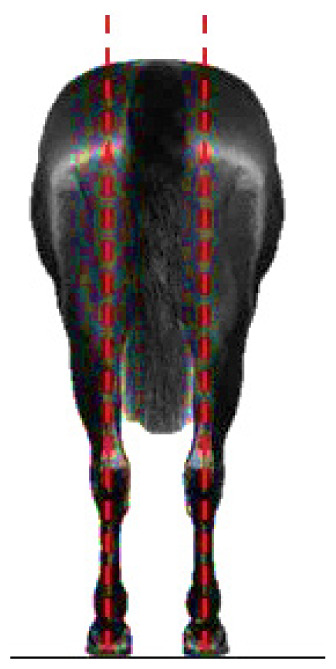	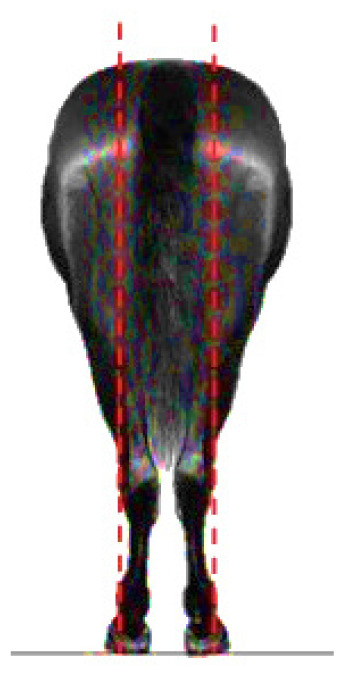

**Table 2:** Descriptive statistics for the dressage variables and hock conformation defects analyzed in the Pura Raza Española horse

Variables		N	Mean (SD)	Range	Class 1 N (%)	Class 2 N (%)	Class 3 N (%)
Dressage	Walk	26,512	6.67 (0.005)	1–9	-	-	-
	PPR	43,619	65.84 (5.216)	11.85–98.10	-	-	-
Hocks defects	Closed	43,302	1.32 (0.600)	1–3	32,373 (74.76)	7,952 (18.36)	2,977 (6.88)
	Convergent	55,339	1.93 (0.680)	1–3	14,889 (26.91)	29,501 (53.31)	10,949 (19.79)

SD, standard deviation; PPR, total points per reprise.

**Table 3:** Inbreeding depression regression coefficients for the dressage variables and hock conformation defects in the Pura Raza Española horse

Variables		Function	F	F_6_	F_k_

			b (t-value, p-value)	b (t-value, p-value)	b (t-value, p-value)
Dressage	Walk	*Lm()*	−0.68 (−5.884, <0.001)	−0.74 (−6.073, <0.001)	−0.75 (−4.191, <0.001)
	PPR		−3.51 (−5.744, <0.001)	−4.08 (−6.316, <0.001)	−3.10 (−3.214, 0.001)
Hock defects	Closed	*Polr()*	1.60 (6.597, <0.001)	1.95 (7.262, <0.010)	2.18 (5.951, <0.010)
	Convergent		−0.09 (0.5709, 0.568)	0.43 (2.037, 0.040)	−0.67 (−2.366, 0.020)

*Lm()* and *Polr()* functions for regression models; b: regression coefficients.

PPR, total points per reprise.

**Table 4:** Heritabilities, inbreeding load ratios, and additive, depression load, and residual variances in dressage variables and hock conformation defects in the Pura Raza Española horse

Genetics parameters		Dressage		Hock defects	

		Walk	PPR	Closed	Convergent
h^2^	Mean (SD)	0.02 (0.005)	0.02 (0.003)	0.15 (0.011)	0.22 (0.020)
	Median	0.02	0.01	0.15	0.22
	HPD95	0.009; 0.03	0.001; 0.021	0.12; 0.17	0.19; 0.25
d^2^	Mean (SD)	0.81 (0.035)	0.89 (0.016)	0.21 (0.051)	0.18 (0.050)
	Median	0.82	0.89	0.21	0.18
	HPD95	0.75; 0.88	0.87; 0.93	0.09; 0.29	0.06; 0.25
r	Mean (SD)	0.05 (0.010)	0.04 (0.006)	-	-
	Median	0.05	0.04	-	-
	HPD95	0.03; 0.08	0.02; 0.04	-	-
$\sigma_{u}^{2}$	Mean (SD)	0.06 (0.011)	3.53 (0.466)	0.07 (0.003)	0.13 (0.005)
	Median	0.05	3.50	0.07	0.13
	HPD95	0.04; 0.08	2.68; 4.38	0.06; 0.08	0.12; 0.14
$\sigma_{i}^{2}$	Mean (SD)	2.74 (0.561)	202.71 (40.185)	0.09 (0.032)	0.10 (0.034)
	Median	2.71	199.04	0.09	0.11
	HPD95	1.75; 3.81	127.47; 285.90	0.03; 0.14	0.03; 0.16
$\sigma_{r}^{2}$	Mean (SD)	0.17 (0.016)	5.43 (0.327)	-	-
	Median	0.17	5.43	-	-
	HPD95	0.14; 0.20	4.79; 6.09	-	-
$\sigma_{h}^{2}$	Mean (SD)	0.13 (0.012)	4.79 (0.440)	-	-
	Median	0.13	4.80	-	-
	HPD95	0.10; 0.15	3.94; 5.65	-	-
$\sigma_{e}^{2}$	Mean (SD)	0.28 (0.003)	10.31 (0.079)	0.29 (0.0034)	0.34 (0.004)
	Median	0.28	10.31	0.29	0.34
	HPD95	0.27; 0.28	10.16; 10.46	0.28; 0.30	0.33; 0.35

Values correspond to a horse with an inbreeding value of 10%.

PPR, total points per reprise; *h*^2^, heritabilities; SD, standard deviation; HPD95, highest posterior density at 95%; *d*^2^, inbreeding depression load ratios; r, repeatability; $\sigma_{u}^{2}$, direct additive genetic variances; $\sigma_{i}^{2}$, inbreeding depression load variances; $\sigma_{r}^{2}$, rider random effect; $\sigma_{h}^{2}$, permanent environmental effect; $\sigma_{e}^{2}$, residual variances.

**Table 5:** Pearson correlations between inbreeding depression load values (below diagonal) and number (and proportion, upper diagonal) of coincident animals with inbreeding load values that improve both traits in the Pura Raza Española horse population analysed

Variables		Dressage		Hock defects	

		Walk	PPR	Closed	Convergent
Dressage	Walk		2,030 (0.51)	1,265 (0.32)	924 (0.23)
	PPR	0.45^***^		886 (0.22)	623 (0.16)
Hock defects	Closed	0.07^***^	−0.02^***^		970 (0.24)
	Convergent	0.07^***^	0.06^***^	0.27^***^	

*** p<0.001.

Values in parentheses represent the proportion of animals (relative to the total number analyzed) showing simultaneous improvement in inbreeding depression load for the corresponding pair of traits.

PPR, total points per reprise.

**Table 6:** Expected genetic responses and the increase in response respect to index 1 (in parentheses) for the selection indexes for dressage and hock defects traits in Pura Raza Española horses

Index type	Objective criteria (economic weight)	Selection criteria	Walk	Closed hock
1	EBV	EBV	0.14 (100)	0.39 (100)
2	EBV	IDL	−0.69 (−589.46)	0.10 (−75.27)
3	EBV (90%) and IDL (10%)	EBV and IDL	0.12 (−17.69)	0.36 (−7.91)
3	EBV (80%) and IDL (20%)	EBV and IDL	0.09 (−35.38)	0.33 (−15.82)
3	EBV (70%) and IDL (30%)	EBV and IDL	0.07 (−53.07)	0.30 (−23.73)
3	EBV (60%) and IDL (40%)	EBV and IDL	0.04 (−70.76)	0.26 (−31.64)
3	EBV (50%) and IDL (50%)	EBV and IDL	0.02 (−88.46)	0.23 (−39.55)

Increase in response is calculated respect to index 1 fixed as a relative response of the 100%. Index type 1: using the EBV selection criteria as the selection objective; index type 2: using the EBV as the selection objective and the IDL as selection criteria; and index type 3: combining the EBV and the IDL as objective and selection criteria. Different economic weight vector (p′) were tested for index 3. For each index, expected genetic response (EGR) were computed, as described in [23].

Values in parentheses indicate the relative increase or decrease in the expected genetic response with respect to Index 1, which was set as the reference (100%).

EBV, estimated breeding values; IDL, *inbreeding depression load*.

## Data Availability

Data are available at https://zenodo.org/records/15362765

## References

[1] Charlesworth D Willis JH The genetics of inbreeding depression Nat Rev Genet 2009 10 783 96 10.1038/nrg2664 19834483

[2] Santana ML Jr Oliveira PS Eler JP Gutiérrez JP Ferraz JBS Pedigree analysis and inbreeding depression on growth traits in Brazilian Marchigiana and Bonsmara breeds1 J Anim Sci 2012 90 99 108 10.2527/jas.2011-4079 21841079

[3] Leroy G Inbreeding depression in livestock species: review and meta-analysis Anim Genet 2014 45 618 28 10.1111/age.12178 24975026

[4] Perdomo-González DI Molina A Sánchez-Guerrero MJ Bartolomé E Varona L Valera M Genetic inbreeding depression load for fertility traits in Pura Raza Española mares J Anim Sci 2021 99 skab316 10.1093/jas/skab316 34718615 PMC8645228

[5] Gómez MD Valera M Molina A Gutiérrez JP Goyache F Assessment of inbreeding depression for body measurements in Spanish Purebred (Andalusian) horses Livest Sci 2009 122 149 55 10.1016/j.livsci.2008.08.007

[6] Poyato-Bonilla J Perdomo-González DI Sánchez-Guerrero MJ Genetic inbreeding depression load for morphological traits and defects in the Pura Raza Española horse Genet Sel Evol 2020 52 62 10.1186/s12711-020-00582-2 33081691 PMC7576714

[7] García-Cortés LA Martínez-Ávila JC Toro MA Fine decomposition of the inbreeding and the coancestry coefficients by using the tabular method Conserv Genet 2010 11 1945 52 10.1007/s10592-010-0084-x

[8] Varona L Altarriba J Moreno C Martínez-Castillero M Casellas J A multivariate analysis with direct additive and inbreeding depression load effects Genet Sel Evol 2019 51 1 12 10.1186/s12711-019-0521-3 31878872 PMC6933709

[9] Meuwissen THE Luo Z Computing inbreeding coefficients in large populations Genet Sel Evol 1992 24 305 13 10.1186/1297-9686-24-4-305

[10] Kalinowski ST Hedrick PW Miller PS Inbreeding depression in the Speke’s gazelle captive breeding program Conserv Biol 2000 14 1375 84 10.1046/j.1523-1739.2000.98209.x

[11] Doekes HP Curik I Nagy I Farkas J Kövér G Windig JJ Revised calculation of Kalinowski’s ancestral and new inbreeding coefficients Diversity 2020 12 155 10.3390/d12040155

[12] Perdomo-González DI Sánchez-Guerrero MJ Molina A Valera M Genetic structure analysis of the Pura Raza Español horse population through partial inbreeding coefficient estimation Animals 2020 10 1360 10.3390/ani10081360 32781594 PMC7459874

[13] Ripollés-Lobo M Perdomo-González DI Sánchez-Guerrero MJ Bartolomé E Valera M Genetic relationship between free movement and under rider gaits in young Pura Raza Española horses Livest Sci 2022 263 105031 10.1016/j.livsci.2022.105031

[14] Ripollés-Lobo M Perdomo-González DI Azor PJ Valera M Evaluation of potential effects and genetic parameters in conformational limb defects in Pura Raza Española horses Ital J Anim Sci 2023 22 407 17 10.1080/1828051X.2023.2206419

[15] Gutiérrez JP Goyache F A note on ENDOG: a computer program for analysing pedigree information J Anim Breed Genet 2005 122 172 6 10.1111/j.1439-0388.2005.00512.x 16130468

[16] Lacy RC Alaks G Walsh A Hierarchical analysis of inbreeding depression in Peromyscus polionotus Evolution 1996 50 2187 200 10.1111/j.1558-5646.1996.tb03609.x 28565659

[17] Rodrigáñez J Toro MA Rodriguez MC Silió L Effect of founder allele survival and inbreeding depression on litter size in a closed line of large white pigs Anim Sci 1998 67 573 82 10.1017/S1357729800033014

[18] Casellas J On individual-specific prediction of hidden inbreeding depression load J Anim Breed Genet 2018 135 37 44 10.1111/jbg.12308 29230876

[19] Caballero A Toro MA Interrelations between effective population size and other pedigree tools for the management of conserved populations Genet Res 2000 75 331 43 10.1017/S0016672399004449 10893869

[20] R Core Team R: a language and environment for statistical computing [Internet] R Foundation for Statistical Computing c2024 [Cited 2025 Apr 1] Available from: https://www.R-project.org/

[21] Hazel LN Lush JL The efficiency of three methods of selection J Hered 1942 33 393 9 10.1093/oxfordjournals.jhered.a105102

[22] Gutiérrez JP Cervantes I Pérez-Cabal MA Burgos A Morante R Weighting fibre and morphological traits in a genetic index for an alpaca breeding programme Animal 2014 8 360 9 10.1017/S1751731113002358 24423382

[23] Perdomo-González DI Sánchez-Guerrero MJ Bartolomé E dos Santos RG Molina A Valera M Designing an early selection morphological traits index for reproductive efficiency in Pura Raza Española mares J Anim Sci 2024 102 skad409 10.1093/jas/skad409 38118055 PMC10762892

[24] Wallin L Strandberg E Philipsson J Genetic correlations between field test results of Swedish warmblood riding horses as 4-year-olds and lifetime performance results in dressage and show jumping Livest Prod Sci 2003 82 61 71 10.1016/S0301-6226(02)00307-X

[25] Borowska A Lewczuk D Comparison of conformation and movement characteristics in dressage and jumping sport warmblood mares based on point evaluation and linear scoring system Animals 2023 13 3101 10.3390/ani13193101 37835707 PMC10571798

[26] Sánchez-Guerrero MJ Cervantes I Molina A Gutiérrez JP Valera M Designing an early selection morphological linear traits index for dressage in the Pura Raza Español horse Animal 2017 11 948 57 10.1017/S1751731116002214 27839527

[27] Solé M Santos R Gómez MD Galisteo AM Valera M Evaluation of conformation against traits associated with dressage ability in unridden Iberian horses at the trot Res Vet Sci 2013 95 660 6 10.1016/j.rvsc.2013.06.017 23880096

[28] Ripollés-Lobo M Perdomo-González DI Valera M Gómez MD Conformational defects in the limbs of Menorca purebred horses and their relationship to functionality Animals 2024 14 1071 10.3390/ani14071071 38612310 PMC11011047

[29] Todd ET Ho SYW Thomson PC Ang RA Velie BD Hamilton NA Founder-specific inbreeding depression affects racing performance in Thoroughbred horses Sci Rep 2018 8 6167 10.1038/s41598-018-24663-x 29670190 PMC5906619

[30] Sairanen J Nivola K Katila T Virtala AM Ojala M Effects of inbreeding and other genetic components on equine fertility Animal 2009 3 1662 72 10.1017/S1751731109990553 22443550

[31] Laseca N Ziadi C Perdomo-Gonzalez DI Valera M Demyda-Peyras S Molina A Reproductive traits in Pura Raza Española mares manifest inbreeding depression from low levels of homozygosity J Anim Breed Genet 2024 141 453 64 10.1111/jbg.12856 38299872

[32] Hill EW Stoffel MA McGivney BA MacHugh DE Pemberton JM Inbreeding depression and the probability of racing in the Thoroughbred horse Proc R Soc B Biol Sci 2022 289 1 7 10.1098/rspb.2022.0487 PMC9240673 35765835

[33] Doekes HP Bijma P Windig JJ How depressing is inbreeding? A meta-analysis of 30 years of research on the effects of inbreeding in livestock Genes 2021 12 926 10.3390/genes12060926 34207101 PMC8234567

[34] Vicente AA Carolino N Ralão-Duarte J Gama LT Selection for morphology, gaits and functional traits in Lusitano horses: I. genetic parameter estimates Livest Sci 2014 164 1 12 10.1016/j.livsci.2014.01.020

[35] Vicente AA Carolino N Ralão-Duarte J Gama LT Selection for morphology, gaits and functional traits in Lusitano horses: II. fixed effects, genetic trends and selection in retrospect Livest Sci 2014 164 13 25 10.1016/j.livsci.2014.03.017

[36] Vostrý L Čapková Z Přibyl J Mach K Analysis of Czech cold-blooded horses: genetic parameters, breeding value and the influence of inbreeding depression on linear description of conformation and type characters Czech J Anim Sci 2011 56 217 30 10.17221/1430-CJAS

[37] Ducro BJ Koenen EPC van Tartwijk JMFM Bovenhuis H Genetic relations of movement and free-jumping traits with dressage and show-jumping performance in competition of Dutch Warmblood horses Livest Sci 2007 107 227 34 10.1016/j.livsci.2006.09.018

[38] Posta J Komlósi I Mihók S Genetic parameters of Hungarian sport horse. Mare performance tests Anim Sci Pap Rep 2010 28 373 80

[39] Schroderus E Ojala M Estimates of genetic parameters for conformation measures and scores in Finnhorse and Standardbred foals J Anim Breed Genet 2010 127 395 403 10.1111/j.1439-0388.2010.00856.x 20831564

[40] Sánchez Guerrero MJ Cervantes I Valera M Gutiérrez JP Modelling genetic evaluation for dressage in Pura Raza Español horses with focus on the rider effect J Anim Breed Genet 2014 131 395 402 10.1111/jbg.12088 24673743

